# Inhibited Carnitine Synthesis Causes Systemic Alteration of Nutrient Metabolism in Zebrafish

**DOI:** 10.3389/fphys.2018.00509

**Published:** 2018-05-09

**Authors:** Jia-Min Li, Ling-Yu Li, Xuan Qin, Pascal Degrace, Laurent Demizieux, Samwel M. Limbu, Xin Wang, Mei-Ling Zhang, Dong-Liang Li, Zhen-Yu Du

**Affiliations:** ^1^Laboratory of Aquaculture Nutrition and Environmental Health, School of Life Sciences, East China Normal University, Shanghai, China; ^2^Shanghai Key Laboratory of Regulatory Biology, Institute of Biomedical Sciences and School of Life Sciences, East China Normal University, Shanghai, China; ^3^Team Pathophysiology of Dyslipidemia, Faculty of Sciences Gabriel, INSERM UMR1231 “Lipides, Nutrition, Cancer,” Université Bourgogne Franche-Comté, Dijon, France; ^4^Department of Aquatic Sciences and Fisheries Technology, University of Dar es Salaam, Dar es Salaam, Tanzania

**Keywords:** low carnitine zebrafish, mildronate, FA β-oxidation, dyslipidemia, metabolism

## Abstract

Impaired mitochondrial fatty acid β-oxidation has been correlated with many metabolic syndromes, and the metabolic characteristics of the mammalian models of mitochondrial dysfunction have also been intensively studied. However, the effects of the impaired mitochondrial fatty acid β-oxidation on systemic metabolism in teleost have never been investigated. In the present study, we established a low-carnitine zebrafish model by feeding fish with mildronate as a specific carnitine synthesis inhibitor [0.05% body weight (BW)/d] for 7 weeks, and the systemically changed nutrient metabolism, including carnitine and triglyceride (TG) concentrations, fatty acid (FA) β-oxidation capability, and other molecular and biochemical assays of lipid, glucose, and protein metabolism, were measured. The results indicated that mildronate markedly decreased hepatic carnitine concentrations while it had no effect in muscle. Liver TG concentrations increased by more than 50% in mildronate-treated fish. Mildronate decreased the efficiency of liver mitochondrial β-oxidation, increased the hepatic mRNA expression of genes related to FA β-oxidation and lipolysis, and decreased the expression of lipogenesis genes. Mildronate decreased whole body glycogen content, increased glucose metabolism rate, and upregulated the expression of glucose uptake and glycolysis genes. Mildronate also increased whole body protein content and hepatic mRNA expression of mechanistic target of rapamycin (*mtor*), and decreased the expression of a protein catabolism-related gene. Liver, rather than muscle, was the primary organ targeted by mildronate. In short, mildronate-induced hepatic inhibited carnitine synthesis in zebrafish caused decreased mitochondrial FA β-oxidation efficiency, greater lipid accumulation, and altered glucose and protein metabolism. This reveals the key roles of mitochondrial fatty acid β-oxidation in nutrient metabolism in fish, and this low-carnitine zebrafish model could also be used as a novel fish model for future metabolism studies.

## Introduction

Abnormalities in lipid metabolism are associated with metabolic syndromes, and animal models of abnormal lipid metabolism are essential for the relevant research (Lopez-Soriano et al., 1997; Brinton, 2003; Cheung and Sanyal, 2008). L-carnitine is a key factor in long chain fatty acid (LCFA) β-oxidation (Bremer, 1983; Bieber, 1988; Zammit et al., 2009), and the L-carnitine deficiency may disrupt lipid catabolism in humans and mice (Karpati et al., 1975; Treem et al., 1988; Kuwajima et al., 1991). Till now, some low-carnitine animal models have been established and applied in the studies of lipid metabolism. However, some of these animal models are difficult to obtain, such as the juvenile visceral steatosis mice some week after birth (Horiuchi et al., 1994). Moreover, some low-carnitine animals have adverse side effects, for example the low-carnitine mammals induced by sodium pivalate would have heart dysfunction (Makrecka et al., 2011).

Mildronate (3-(2,2,2-trimethylhydrazinium) propionate) is a structural analog of the precursor to L-carnitine synthesis (γ-butyrobetaine). It can inhibit endogenous carnitine biosynthesis (Simkhovich et al., 1988) and carnitine re-absorption in kidneys (Kuwajima et al., 1999), thereby decreasing the carnitine concentrations in heart, liver, skeletal muscle, and other organs (Spaniol et al., 2001; Liepinsh et al., 2006). Mildronate was first fed to rats in 1986 to decrease the myocardium carnitine concentration to establish a low-carnitine animal model (Simkhovich et al., 1986). In the mildronate-induced low-carnitine mammalian models, mildronate directly inhibits LCFA β-oxidation by inhibiting the transport of FAs from cytosol into the mitochondrial matrix (Zhv et al., 1989), and regulates energy metabolism by facilitating glycolysis (Liepinsh et al., 2008, 2009). These properties make mildronate a useful treatment for diabetes and angiocardiopathy. Mildronate-treated animals have also been used to study non-alcoholic fatty liver disease (Du et al., 2013) and lipid flux between different tissues (Osmundsen et al., 1998; Degrace et al., 2007; Du et al., 2010). To date, the effects of mildronate administration have been studied in humans, rodents (Dhar et al., 1996), and dogs (Kirimoto et al., 1995) but not in non-mammalian animals such as fish.

At present, severe fat accumulation is common among farmed fish and causes a number of adverse effects on their growth and health (Du et al., 2008; Yan et al., 2015). Although many fish physiologists suppose that the severe fat accumulation is also correlated with impaired mitochondrial fatty acid β-oxidation, scientists have been unable to fully determine the metabolic causes of this dyslipidemia, in part because of the lack of fish models of dyslipidemia. Therefore, mildronate could be a useful tool to artificially decrease the endogenous carnitine concentration and could be used to establish a low-carnitine fish model with impaired mitochondrial fatty acid β-oxidation.

The zebrafish has been used as a model to investigate the molecular mechanisms in fish nutrition (Ulloa et al., 2011; Fang et al., 2014). In the present study, zebrafish was chosen to establish a low-carnitine model by dietary mildronate supplementation, and the biochemical and molecular characteristics of lipid, carbohydrate, and protein metabolism were systemically evaluated. The objective of this study was to illustrate the alteration of metabolism caused by impaired mitochondrial fatty acid β-oxidation, which was induced by carnitine synthesis inhibition. This could help fish physiologists to identify the causes of abnormal metabolic status, such as severe fat accumulation, and provide basic metabolic information on this low-carnitine model.

## Materials and Methods

### Animal Ethics

All experiments were conducted under the Guidance of the Care and Use of Laboratory Animals in China. This study was approved by the Committee on the Ethics of Animal Experiments of East China Normal University.

### Fish, Diets, and Sampling

Male zebrafish (0.25 ± 0.03 g) were purchased from Chinese National Zebrafish Resource Center (Wuhan, China). Before experiments, fish were acclimated for 1 week, during which they were fed on a commercial zebrafish diet (protein ≥50%, lipid ≥8%, nutrient composition shown in the diet label, Shengsuo Co., Shandong, China). Before the feeding trial, the dough particle containing mildronate or not and the basic diet were prepared. Mildronate diet was prepared by dissolving mildronate into distilled water, and the mildronate solution was mixed with given amount of wheat flour to make wet dough. Mildronate was added into the dry wheat flour-dough particles to ensure the final designed intake doses were achieved when the dough particles were fed at 1% BW. The wet dough was dried at 60°C for 8 h, and stored at –20°C until needed for feeding. The basal diet and dough particles were pelleted into proper sizes (about 0.2 mg per particle) for zebrafish feeding. The formulations of the basal diet and wheat flour-dough particle are listed in **Supplementary Table S1**. After acclimation, the first experiment was conducted to evaluate the time and dose-dependent carnitine-lowering effects of mildronate. In this experiment, 360 zebrafish were randomly divided into four groups (three tanks per group, 30 fish per tank) and fed with different mildronate doses (0, 100, 500, 1000 mg/kg BW). Every morning, the dough particles containing different concentrations of mildronate or not were first fed to the mildronate group and control group, respectively, at 1% BW. Afterward, both groups were fed with the basic diet at 3% BW. After 1 and 3 weeks, the livers and muscles of at least 30 fish (10 fish pertank) were taken for measurements of carnitine and mildronate. During the experiment, the photoperiod was 12 h/12 h, and the temperature was kept at 26 ± 2°C. The weight of the fish in each tank was recorded every one week, and the feeding amount was adjusted correspondingly. The first experiment was conducted for 3 weeks.

The second experiment was conducted in order to study the metabolic characteristics of low carnitine induced by mildronate at similar conditions to the first experiment. To achieve this, 360 zebrafish were randomly divided into two groups (three tanks per group and 60 fish per tank). The mildronate dose was set at 0.05% BW (500 mg/kg BW) which was an effective dose that reduced carnitine for at least 3 weeks determined in the first experiment. The feeding strategy was the same as that in the first experiment. After 7 weeks, the whole experiment was terminated. At the end of the experiment, the 12-h fasted fish in each tank were anesthetized in ice water by following the standard protocol (Matthews et al., 2002), and sampled for liver, muscle, and visceral adipose tissue (VAT) collections, for molecular and biochemical assays.

### Carnitine and Mildronate Concentration Determination

After the feeding trial, the whole liver and parts of muscle of six fish were collected from each group, weighted and homogenized by distilled water (1:10, w:v). The tissue homogenate was centrifuged at 2000 rpm/min for 15 min. A 100 μL aliquot of tissue homogenate supernatant was first incubated by using 50 μL KOH (1 mol/L) at 37°C for 30 min to fully hydrolyze the combined carnitine, and 10 μL HCl (1 mol/L) was then added to neutralize the solution (sample A). A total of 60 μL distilled water was added directly into another 100 μL aliquot of tissue homogenate (sample B). A total of 60 μL distilled water was added directly into third 100 μL aliquot of tissue homogenate (sample C). Protein in sample A, B, and C was removed by precipitating with 200 μL of cold acetonitrile (ACN; containing 1 μg/mL carbachol, the internal standard, IS) and centrifuging at 16,900 × *g* for 20 min. One microliter of the supernatant was injected for LC-MS/MS analysis. Total carnitine was defined as the concentration of carnitine in sample A, free carnitine was defined as the concentration of carnitine in sample B, and mildronate was defined using sample C. The details of LC-MS measurements were presented by our recent publication (Li et al., 2017), and the multiple reaction monitoring (MRM) mode monitoring the transitions of m/z 147.2→58.2 for mildronate was added in the present study.

### Mitochondrial and Peroxisomal [1-^14^C] Palmitate Oxidation in Liver and Muscle

After the feeding trial, the whole liver and parts of muscle of six fish were collected from each group, weighted, and homogenized in the ice-cold 0.25 M-sucrose medium containing 2 mM-EGTA and 10 mM-Tris–Cl, pH 7.4 (1:40 and 1:10, w:v) by using a drill-driven Teflon glass homogenizer with 3–4 strokes. The samples of homogenates were used for the immediate measurement of mitochondrial and peroxisomal [1-^14^C] palmitate β-oxidation. Palmitate oxidation rates were detected at 28°C using two media described earlier (Veerkamp et al., 1983), the first media containing exogenous L-carnitine allowing both mitochondrial and peroxisomal β-oxidation to occur, and the second one allowing peroxisomal β-oxidation only. After 0.5 h, the radio activity initially held by [1-^14^C] palmitate was recovered on labeled short molecules released from the β-oxidative cycle and soluble in perchloric acid (acid-soluble products, ASP). The pure radioactive ASP medium was collected using 0.45 μm membrane filters and measured after mixing with the scintillation cocktail in a liquid scintillation spectrometer (Du et al., 2006). In order to measure the β-oxidation activities of the original tissues of fish, parallel assays without exogenous L-carnitine in the reaction media were also performed.

### The *in Vivo* Metabolic Rates of Fatty Acid, Glucose, and Amino Acids

After the feeding trial, 24 fish from each group were euthanized (MS-222, 20 mg/L, Welker et al., 2007) and eight fish were injected intraperitoneal (i.p.) with 5 μL of DMSO contain palmitic acid (final concentration with 1.25 μmol per 1 g BW). Another 16 fish were divided as two groups (eight fish per group) and were injected i.p. with 5 μL of PBS containing glucose (final concentration was 5.5 μmol per BW) and 5 μL of PBS contain amino acid mixture (L-Lys:L-Arg: DL-Met:L-His:L-Val, 2.7:2:1:1:1.3) (final concentration with 1.25 μmol per BW), respectively. The injected fish were immediately moved into a two-channel oxygen meter (782 Oxygen Meter, Strathkelvin Instruments Limited, North Lanarkshire, United Kingdom) to measure the oxygen consumption rates. In the assay, the fish were placed in a chamber with 0.65 L air-saturated water at a stable 24°C for 10 min. The metabolic rates of fatty acid, glucose, and AA mixture were calculated as: M_O2_ = [ΔO_2_ concentration (mg/L) × water volume (L)]/[fish mass (g) × time (h)].

### Biochemical Composition of Whole Fish and Tissues

The crude lipid of the whole fish body was extracted and measured using methanol and chloroform (1:2, v:v) as previously described (Folch et al., 1957; Lambert and Dehnel, 1974). Whole fish glycogen and protein were assessed by the commercial kit (Jiancheng Biotech Co., China) and by Kjeltec^TM^ 8200 (FOSS, Sweden), respectively. TG content of tissues was measured by thin layer chromatography (TLC; Cejas et al., 2003; Aurousseau et al., 2004). Briefly, total lipid of tissues was extracted using methanol and chloroform (1:2, v:v), the TG was separated from the total lipid by TLC using hexane, diethyl ether, and glacial acetic acid (80:20:2, v:v:v) as the mobile phase, and scanned and quantified by KH-3000 TLC Scanner (Kezhe, Shanghai, China).

### Histological Analysis

Pieces of liver (5 mm × 5 mm) were fixed in 4% paraformaldehyde and embedded in paraffin as described (Betancor et al., 2015). Sections of 5 μm thickness were stained with hematoxylin and eosin (H & E), observed and photographed by Olympus BX53.

### Quantitative Real-Time PCR

Total RNA was isolated by using a Tri Pure Reagent (Aidlab, China). The quality and quantity of total RNA were tested by NANODROP 2000 Spectrophotometer (Thermo, United States). cDNAs of tissues total RNA were synthesized using a PrimerScript^TM^ RT reagent Kit with a gDNA Eraser (Perfect Real Time; Takara, Japan) by S1000^TM^ Thermal Cycler (Bio-Rad, United States). Elongation factor 1 alpha (*ef1α*) and *β-actin* were used as the reference genes. The primers of all testing genes for Quantitative PCR (qPCR) were listed in **Supplementary Table S2**. The qPCR assay was carried out as described previously (Li et al., 2017).

### Statistical Analyses

All results are presented as mean ± SEM. In the data comparison among different mildronate concentrations, the data of different groups were firstly subjected to one-way ANOVA. When significant difference (*P* < 0.05) was found, a Duncan’s multiple range test was used to estimate the differences. Independent-samples *t*-test was performed to evaluate the significant difference (*P* < 0.05) of variables between control and mildronate group. All analyses were conducted using the SPSS Statistics 19.0 software (IBM, United States).

## Results

### Mildronate Decreased Endogenous Carnitine Concentrations

**FIGURE 1:**
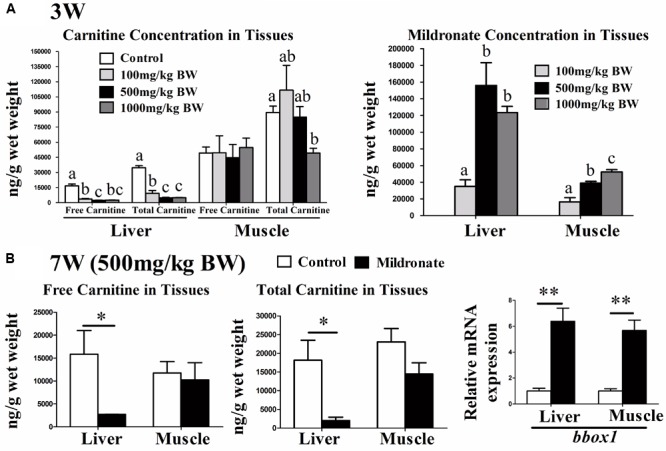
The effect of the dietary mildronate on establishing the low-carnitine zebrafish. **(A)** The free carnitine, total carnitine and mildronate concentration in liver and muscle after 3 weeks of the first experiment; **(B)** the free and total carnitine, and the mRNA expression of carnitine synthesis (*bbox1*) in liver and muscle after 7 week of the second experiment. All values are means ± SEM (*n* = 6). In A, values that are not followed by the same letter are significantly different (*P* < 0.05). In B, values with ^∗^ and ^∗∗^ statistically differ at *P* < 0.05 and *P* < 0.01, respectively. *bbox1*, butyrobetaine (gamma), 2-oxoglutarate dioxygenase (gamma-butyrobetaine hydroxylase) 1.

Before the 7-week feeding trial, the time and dose-dependent effects of mildronate on carnitine concentrations in zebrafish were evaluated. None of the three doses of mildronate trialed (100, 500, and 1000 mg/kg BW) decreased the carnitine concentrations in zebrafish during the first week (data not shown). After 3 weeks, the 100, 500, and 1000 mg/kg BW doses of mildronate decreased the hepatic concentration of free carnitine by 78.6% (16738 vs. 3585.87 ng/g), 86.0% (16738 vs. 2334.93 ng/g), and 84.6% (16738 vs. 2578.27 ng/g), respectively, and the hepatic concentration of total carnitine by 73.2% (34661.2 vs. 9283.8 ng/g), 86.1% (34661.20 vs. 4803.73 ng/g), and 85.4% (34661.2 vs. 5043.13 ng/g), respectively (**Figure 1A**). In muscle, after 3 weeks the three mildronate doses did not affect the free carnitine concentration, and only the 1000 mg/kg BW dose decreased the total carnitine concentration, which showed a 44.9% (89533 vs. 49332 ng/g) reduction (**Figure 1A**). The deposition of mildronate in liver indicated that 500 and 1000 mg/kg BW doses significantly increased mildronate deposition as compared with the 100 mg/kg BW doses, but no significant differences were found between 500 and 1000 mg/kg BW doses (**Figure 1A**). In muscle, the deposition of mildronate showed a dose-dependent increase, and the significant differences were found among the three mildronate doses. These results indicate that mildronate accumulates in liver more than muscle, and has a greater carnitine lowering effect in liver than muscle. Although the mildronate doses of 500 and 1000 mg/kg BW would significantly decreased carnitine concentrations in liver and muscle, further testing indicated that, compared to the control, the dose of 1000 mg/kg caused significantly higher mRNA expression of cytochrome P450 enzyme 1 (*cyp1*), which is a detoxification marker, in muscle and colony stimulating factor (*csf*), which is an inflammatory marker, in liver (**Supplementary Figure S1**). However, 500 mg/kg BW mildronate treatment didn’t change these gene expressions. This indicated the dose of 1000 mg/kg mildronate would have potential toxic effects in impairing fish health. From these data, we predicted that a mildronate-induced low-carnitine zebrafish model could be established by feeding the fish with 500 mg/kg BW mildronate for at least 3 weeks.

At the end of the 7-week feeding trial using 500 mg/kg BW/d mildronate (mildronate group), the fish were in good health visually and had similar BWs and survival rates as the untreated controls (data not shown). The mRNA expressions of several inflammatory factors [(interleukin 1 beta) *il1b*, (tumor necrosis factor α) *tnfa* and (transforming growth factor β1) *tgfb1*, (macrophage migration inhibitory factor) *mif*, (interferon γ) *ifn-γ, csf*) and some detoxification or apoptosis markers (*cyp1*), (heat shock protein 70) *hsp70*, (caspase 3) *casp3*] were also comparable between the mildronate and control groups (**Supplementary Figures S2A,B**), suggesting that mildronate treatment for 7 weeks does not cause severe adverse effects on fish health. As shown in **Figure 1B**, the free and total carnitine concentrations in liver of the mildronate group were significantly lower than those of the control group (*P* < 0.05). The mRNA expression of butyrobetaine (gamma), 2-oxoglutarate dioxygenase (gamma-butyrobetaine hydroxylase) 1 (*bbox1*), a key enzyme in carnitine synthesis, was significantly increased in liver and muscle of the mildronate group (*P* < 0.01), which could be a compensatory response to the lower endogenous carnitine content.

### Mildronate Specifically Caused Lipid Accumulation in Liver by Inhibiting the FA β-Oxidation Reaction but Increased β-Oxidation Enzymic Capability

**FIGURE 2:**
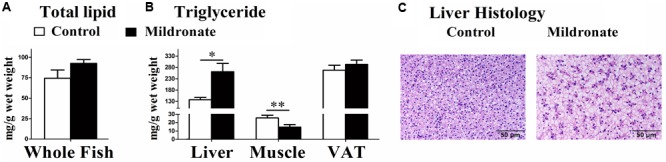
The effect of the dietary mildronate on lipid deposition in whole body and tissues. **(A)** The total lipid content in the whole fish; **(B)** the TG concentration in liver, muscle and VAT (visceral adipose tissue); **(C)** the liver histological characteristics (HE staining). All values are means ± SEM (*n* = 6). Values with ^∗^ and ^∗∗^ statistically differ at *P* < 0.05, and *P* < 0.01, respectively.

As shown in **Figure 2A**, the whole fish lipid content in mildronate-treated zebrafish trended to be higher than the control, but no significant difference was found (*P* > 0.05). The hepatic concentration of TG was significantly higher in the mildronate group than the control group (*P* < 0.05), whereas the TG concentration in muscle was decreased in the mildronate group (*P* < 0.01) (**Figure 2B**). The VAT TG concentrations were comparable between the mildronate and control groups. Histological images of the liver clearly indicated that the mildronate-treated zebrafish accumulated more fat in liver than the control group (**Figure 2C**).

**FIGURE 3:**
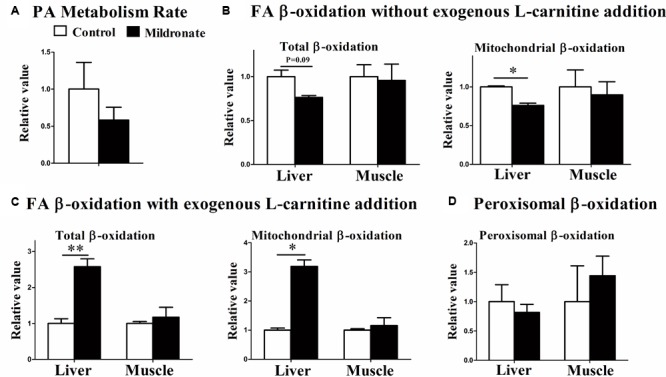
The effect of the dietary mildronate on the FA metabolism rate and the FA β-oxidation in tissues. **(A)** Palmitic acid metabolism rate; **(B)** FA β-oxidation without exogenous L-carnitine addition; **(C)** FA β-oxidation with exogenous L-carnitine addition; **(D)** the peroxisomal β-oxidation. All values are means ± SEM (*n* = 4). Values with ^∗^ and ^∗∗^ statistically differ at *P* < 0.05, and *P* < 0.01, respectively.

To evaluate actual FA degradation *in vivo*, the fish were injected with palmitic acid and the respiration rate (metabolism rate) was measured. The data indicated that the FA metabolism rate in the mildronate group tended to decrease as compared with the control group, but no significant difference was found between both groups (**Figure 3A**). [1-^14^C] palmitic acid β-oxidation was further measured *in vitro* using liver and muscle homogenates with or without exogenous L-carnitine addition. Compared with control fish, in the actual state (without exogenous L-carnitine addition), mitochondrial and total FA β-oxidation efficiencies were decreased in the liver of mildronate-treated fish, but the significant difference was only found in the mitochondrial FA β-oxidation efficiencies (*P* < 0.05; **Figure 3B**). No changes were found in the FA β-oxidation efficiencies in muscle (**Figure 3B**). Conversely, when exogenous L-carnitine was added to the *in vitro* reaction system, mitochondrial and total FA β-oxidation efficiencies showed a 1.5-fold increase in the liver of mildronate-treated fish compared with control fish (total β-oxidation *P* < 0.01, mitochondrial β-oxidation *P* < 0.05) but showed no differences (*P* > 0.05) between groups in muscle (**Figure 3C**). The efficiency of peroxisomal FA β-oxidation, which does not require L-carnitine, was comparable between the two groups, without any significant difference between them (*P* > 0.05; **Figure 3D**). These findings indicate that in the low hepatic carnitine state the efficiency of mitochondrial β-oxidation was reduced, but the activities of related enzymes were elevated as a compensatory response.

### Mildronate Treatment Changed the Expression of Lipid Metabolism-Related Genes

**FIGURE 4:**
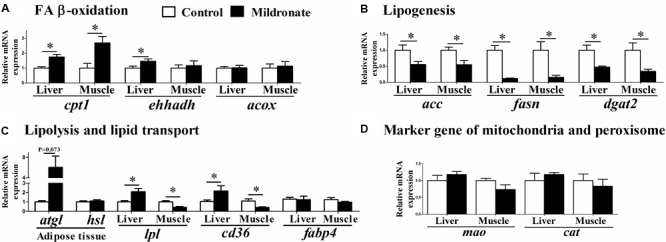
The effect of the dietary mildronate on the mRNA expression of lipid metabolism. **(A)** FA β-oxidation related genes; **(B)** lipogenesis related genes; **(C)** lipolysis and lipid transport related genes; **(D)** marker gene of mitochondria and peroxisome. All values are means ± SEM (*n* = 6). Values with ^∗^ and ^∗∗^ statistically differ at *P* < 0.05, and *P* < 0.01, respectively. *cpt1*, carnitine palmitoyltransferase 1; *ehhadh*, enoyl-CoA, hydratase/3-hydroxyacyl CoA dehydrogenase; *acox*, acyl-CoA oxidase; *acc*, acetyl-CoA carboxylase; *fasn*, fatty acid synthase; *dgat2*, diacylglycerol O-acyltransferase 2; *lpl*, lipoprotein lipase; *atgl*, adipose triglyceride lipase; *hsl*, hormone-sensitive lipase; *fabp4*, fatty acid binding protein 4; *cd36 (fatp)*, fatty acid transport protein; *mao*, monoamine oxidase; *cat*, catalase.

Mildronate significantly increased the mRNA expression of carnitine palmitoyltransferase 1 (*cpt1*; *P* < 0.05), the rate-limiting enzyme of mitochondrial β-oxidation in the liver and muscle (**Figure 4A**). Likewise, mildronate increased the mRNA expression of enoyl-CoA, hydratase/3-hydroxyacyl CoA dehydrogenase (*ehhadh*) in the liver (*P* < 0.05). No differences were found in acyl-CoA oxidase (*acox*) mRNA expression between groups (*P* > 0.05). Among the lipogenesis-related genes (**Figure 4B**), acetyl-CoA carboxylase (*acc*), fatty acid synthase (*fasn*), and diacylglycerol O-acyltransferase 2 (*dgat2*) mRNA expression were significantly decreased in the liver and muscle of the mildronate group (*P* < 0.05). Among the lipid transport-related genes, lipoprotein lipase (*lpl*) and FA transport protein (*cd36*) mRNA expression were significantly increased in the mildronate group in liver (*P* < 0.05), but decreased in the muscle of the mildronate group (*P* < 0.05) (**Figure 4C**). The mRNA expression of adipose triglyceride lipase (*atgl*) tended to increase in the mildronate group in VAT, although there was no significant difference as compared with the control (*P* = 0.073). As marker genes of mitochondria and peroxisomes, monoamine oxidase (*mao*) and catalase (*cat*) showed no differences (*P* > 0.05) in mRNA expression between groups in either nutritional state (**Figure 4D**).

### Mildronate Improved Glucose Metabolism

**FIGURE 5:**
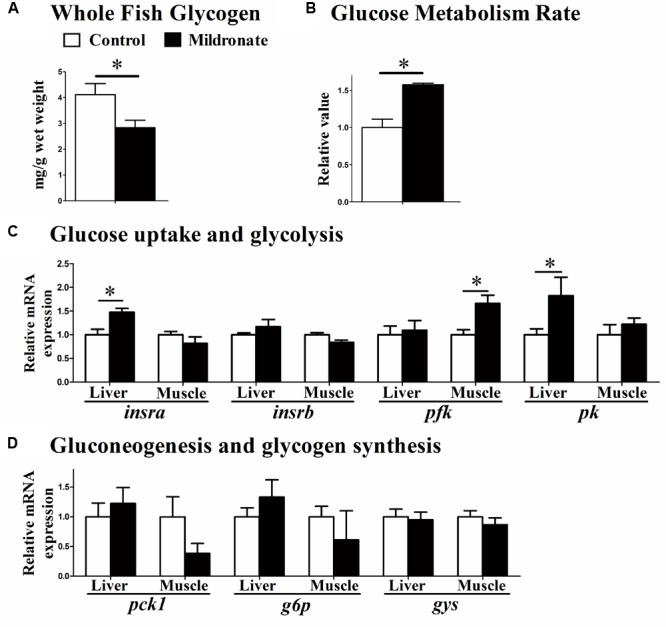
The effect of the dietary mildronate on glucose metabolism. **(A)** Whole fish glycogen; **(B)** glucose metabolism rate; **(C)** glucose uptake and glycolysis related genes; **(D)** gluconeogenesis and glycogen synthesis related genes. All values are means ± SEM (*n* = 6), except the **(B)** (*n* = 4). Values with ^∗^ statistically differ at *P* < 0.05. *insra*, insulin receptor a; *insrb*, insulin receptor b; *pfk*, phosphofructokinase; *pk*, pyruvate kinase; *pck1*, phosphoenolpyruvate carboxykinase 1; *g6p*, glucose-6-phosphatase; *gys*, glycogen synthase.

Whole body glycogen concentrations were decreased in the mildronate-treated fish (*P* < 0.05; **Figure 5A**), and their respiration rates were increased after a glucose injection (*P* < 0.05; **Figure 5B**), indicating that glucose metabolism rate was increased by mildronate. The mRNA expression of insulin receptor a (*insra*) was higher in the liver of the mildronate group than the control group (*P* < 0.05), though was comparable between groups in muscle (*P* > 0.05; **Figure 5C**). No differences were found in insulin receptor b (*insrb*) mRNA expression between groups (*P* > 0.05). The mRNA expression of muscle phosphofructokinase (*pfk*;*P* < 0.05) and liver pyruvate kinase (*pk*; *P* < 0.05) was increased in the mildronate group (**Figure 5C**). Among the genes related to gluconeogenesis and glycogen synthesis (**Figure 5D**), there were no significant differences (*P* > 0.05) between groups in the mRNA expression of phosphoenolpyruvate carboxykinase 1 (*pck1*), glucose-6-phosphatase (*g6p*) or glycogen synthase (*gys*) in the liver and muscle of mildronate-treated fish.

### Mildronate Affected Protein Metabolism

**FIGURE 6:**
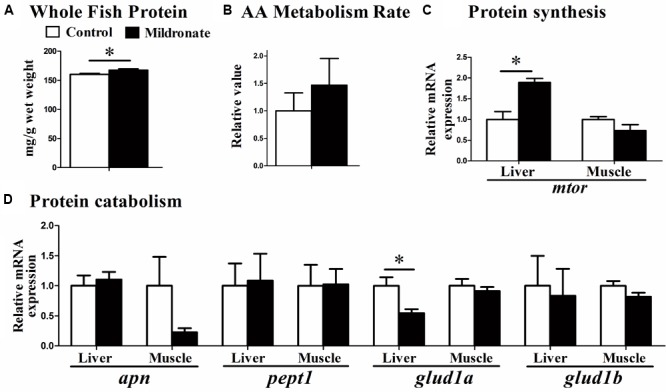
The effect of the dietary mildronate on protein metabolism. **(A)** Whole fish protein; **(B)** AA mixture metabolism rate; **(C)** protein synthesis related gene; **(D)** protein catabolism related genes. All values are means ± SEM (*n* = 6), except the **(B)** (*n* = 4). Values with ^∗^ and ^∗∗^ statistically differ at *P* < 0.05, and *P* < 0.01, respectively. *apn*, aminopeptidase n; *pept1*, peptide transporter 1; *glud1a*, glutamate dehydrogenase 1 a; *glud1b*, glutamate dehydrogenase 1 b; *mtor*, mechanistic target of rapamycin.

Whole body protein concentrations were significantly increased in mildronate group (*P* < 0.05; **Figure 6A**). However, metabolism rate after the injection of an amino acid mixture was comparable between groups (*P* > 0.05; **Figure 6B**). Among the genes related to amino acids and protein synthesis (**Figure 6C**) and catabolism (**Figure 6D**), a lower mRNA expression of glutamate dehydrogenase 1a (*glud1a*; *P* < 0.05), which is related to amino acid catabolism, and a higher expression of *mtor* were found in the liver of the mildronate group (*P* < 0.05). There were no significant differences (*P* > 0.05) in gene expression between groups in aminopeptidase n (*apn*), peptide transporter 1 (*pept1*), or glutamate dehydrogenase 1b (*glud1b*).

## Discussion

### Establishment of a Mildronate-Induced Low-Carnitine Zebrafish Model

One purpose of the present study was to establish a mildronate-induced low-carnitine zebrafish model. In the dose experiment, a dietary mildronate dose of 500 mg/kg BW/d was the most suitable for lowering the endogenous carnitine concentration of zebrafish (mainly in the liver) within 3 weeks. After 7 weeks of mildronate treatment at this dosage, the free and total carnitine concentrations in zebrafish liver were markedly decreased (by 82.9% and 88.8%, respectively) showing that an appropriate mildronate dosage can be used to establish a low-carnitine fish model. However, compared with liver, muscle carnitine concentrations were not affected by dietary mildronate in zebrafish, likely attributable to the lower deposition of mildronate in muscle than liver. Notably, the dose of 1000 mg/kg mildronate could significantly lowered carnitine concentration in muscle in 3 weeks (**Figure 1**), but also caused higher expressions in the stress and toxic genes (**Supplementary Figure S1**). Therefore, the present study also indicates that the effect of dietary mildronate in lowering endogenous carnitine concentrations in zebrafish was dose and time dependent and tissue specific. In mammals, previous studies have shown that carnitine concentrations can be effectively lowered by mildronate in many tissues, including heart (Tsoko et al., 1995; Hayashi et al., 2000; Liepinsh et al., 2006), liver (Simkhovich et al., 1988; Tsoko et al., 1995; Hayashi et al., 2000; Spaniol et al., 2001; Degrace et al., 2007), skeletal muscle (Tsoko et al., 1995; Spaniol et al., 2001), brain (Tsoko et al., 1995; Spaniol et al., 2001), testis (Dambrova et al., 2008), and kidney (Tsoko et al., 1995). Although the toxic effects of different mildronate doses were not commonly evaluated in these mammalian studies, the mildronate-induced carnitine lowering effect is still likely to be more tissue-specific in fish than mammals.

Mildronate acts by lowering endogenous carnitine concentrations and inhibiting mitochondrial FA β-oxidation. A previous study in mammals showed that mitochondrial FA β-oxidation is not efficiently inhibited when the endogenous carnitine concentration is decreased by 50% (Heinonen and Takala, 1994). Correspondingly, in the present study, because the carnitine concentration was decreased by less than 50% in muscle, the muscle mitochondrial FA β-oxidation was unaffected. However, the depletion of carnitine in liver by 82.9% or more resulted in significantly lower liver mitochondrial FA β-oxidation and increased lipid accumulation in liver. Considering a higher mildronate dose, such as 1000 mg/kg BW, could also efficiently reduce carnitine concentration in muscle, higher mildronate dose might also inhibit muscle mitochondrial FA β-oxidation in zebrafish. Therefore, the inhibition effect of mildronate on tissue mitochondrial FA β-oxidation was highly correlated with the carnitine-lowering effect of mildronate in different tissues. Actually, we also noticed that the mRNA expressions of a number of genes in muscle had been changed by 500 mg/kg mildronate treatment. This suggests that this dose of mildronate could induce changes in gene expression level, but was not enough to trigger significant phenotypes in biochemical or physiological levels in muscle. Nevertheless, this variable carnitine-lowering effect of mildronate on different tissues should be carefully evaluated in further studies.

### Characteristics of Lipid Metabolism in the Low-Carnitine Zebrafish

The main phenotypic characteristics of the mildronate-treated zebrafish were an accumulation of lipid in liver and decreased endogenous carnitine concentrations, with the latter inhibiting FA β-oxidation efficiency. Similarly, studies in mammals have shown that low carnitine concentrations in liver induced by mildronate often lead to increased fat accumulation (Hayashi et al., 2000; Spaniol et al., 2001, 2003; Peschechera et al., 2005) accompanied by decreased FA β-oxidation in liver, heart, or whole body (Simkhovich et al., 1988; Spaniol et al., 2001, 2003; Peschechera et al., 2005). Therefore, mildronate-induced fat accumulation in low-carnitine animals is mainly caused by inhibited FA β-oxidation in mitochondria. However, FA β-oxidation efficiencies increased when exogenous L-carnitine was added to the *in vitro* reaction system (**Figure 3C**), suggesting that the activities of FA β-oxidation-related enzymes increase as a compensatory response to decreased endogenous carnitine concentrations. The increased mRNA expression of *cpt1* in the liver of the mildronate group was another proof for the suggestion. Unlike for liver, the lipid concentration in muscle decreased in the mildronate group (**Figure 2B**), and the degree of decrease in total carnitine concentration in muscle was much less than that in liver. Considering that mRNA expression of lipid transport-related genes (*cd36* and *lpl*) was increased in liver and decreased in muscle in the mildronate-treated zebrafish, we supposed that in the present study, the released lipids from adipose tissue were mainly delivered to liver, which had higher energy requirement than muscle because of the suppression of FA β-oxidation, as previously reported in rats (Degrace et al., 2007). Therefore, compared to the control, relatively less adipose tissue-sourced lipid was delivered to the muscle of mildronate-treated fish and caused the decrease of muscle lipid content. Notably, our parallel experiment in Nile tilapia using 1000 mg/kg mildronate caused decrease of carnitine concentration in muscle, but did not changed the muscle lipid content (data not shown). This not only verified that the higher sensitivity of liver to mildronate than muscle could be a common phenotype in fish, but also suggests that the muscle lipid in the mildronate-treated fish may not be directly correlated with muscle mitochondrial FA β-oxidation efficiency, but is more likely to be affected by the transport of lipid into muscle cells. However, this hypothesis is still waiting for further investigation.

Mitochondria and peroxisome cooperate to regulate FA β-oxidation (Mannaerts et al., 1979; Van den Branden and Vamecq, 2003). In mammals, peroxisomal β-oxidation is strengthened when mitochondrial FA oxidation is inhibited to compensate for the use of long chain fatty acyl-CoA (Tsoko et al., 1995; Spaniol et al., 2003; Peschechera et al., 2005; Degrace et al., 2007). In the present study, peroxisomal β-oxidation did not show a compensatory increase in the mildronate-treated fish in which mitochondrial FA β-oxidation was inhibited. In our recent study (Li et al., 2017), dietary supplementation of L-carnitine increased the endogenous concentration of carnitine and the efficiency of mitochondrial FA β-oxidation, though peroxisomal β-oxidation efficiency did not change. These findings indicate that zebrafish peroxisomes may not be as sensitive to variations in mitochondrial activity as those of mammals. This suggests that impaired mitochondrial function in fish, at least in zebrafish, would cause more severe physiological damage as compared with mammals.

The low hepatic carnitine concentrations inhibited lipid degradation and resulted in insufficient energy supply. The mRNA of ATGL, a key lipolysis enzyme in adipose tissue (Zimmermann et al., 2004), was highly expressed in the mildronate group, which may cause greater release of FA into the circulation. Likewise, the mRNA of lipoprotein lipase (LPL), an enzyme that catalyzes the lipolysis of TG in the very low-density lipoprotein (VLDL) and chylomicrons (Kersten, 2014), was highly expressed in the liver of the mildronate group. In mildronate-treated rats, the mRNA expression of LPL and Cluster determinant 36 (CD36) was upregulated in the heart accompanied by higher serum FA concentrations (Degrace et al., 2004). This may suggest that an insufficient energy supply in liver triggers lipolysis in other tissues to strengthen the uptake of circulating FA into liver. This could have promoted the lipid accumulation in the liver of mildronate-treated fish, and depressed the expression of de novo lipogenesis-related genes in liver. This process has been verified in mildronate-treated rats (Degrace et al., 2007), which showed severe hepatic steatosis accompanied by increased adipose lipolysis and liver lipid uptake, indicating that liver and adipose tissue cooperate to regulate lipid flux. This suggests that mammals and fish may have similar cooperative mechanisms between liver and adipose tissue to regulate lipid flux.

### Effects of Altered Lipid Metabolism on Glucose and Protein Metabolism in the Low-Carnitine Zebrafish

The balance of lipid, glucose, and protein metabolism contributes to maintaining energy homeostasis (Trevisan et al., 1986; Solini et al., 1997; Li et al., 2017). Therefore, a reduction in endogenous carnitine could also affect glucose and protein metabolism.

Previous studies have shown that low-carnitine mammals can improve their glucose utilization by increasing glucose transport and glycolysis rate to compensate the decreasing proportion of energy from FA degradation (Yonekura et al., 2000; Degrace et al., 2007; Liepinsh et al., 2008). In the present study, whole body glycogen content decreased with increasing glucose metabolism rates in the low-carnitine fish, indicating that glucose utilization was also increased to counteract the decrease in FA β-oxidation. Moreover, the mRNA expression of insulin receptor (IR) in liver of the mildronate group was increased. Upregulation of IR is reported to tightly correlate with increased cellular glucose uptake (Bryant et al., 2002; Nevado et al., 2006), thus the higher glucose metabolism rate in the mildronate-treated zebrafish is likely to be related to higher glucose uptake. In addition, glycolysis-related genes (*pfk, pk*) in liver and muscle were upregulated. These results indicate that glycolysis rate was increased in the low-carnitine zebrafish. Correspondingly, our recent fish studies show that improved lipid catabolism, either through peroxisome proliferator-activated receptor α (PPARα) activation or increasing endogenous concentrations of L-carnitine, can decrease the utilization of glucose (Ning et al., 2016; Li et al., 2017). Lipid and glucose metabolism thus appear to cooperate to maintain energy homeostasis, though this process needs further investigation.

**FIGURE 7:**
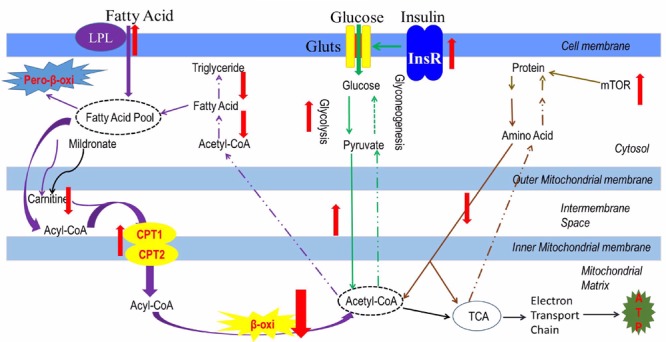
The metabolic characteristics of a low-carnitine zebrafish model. Purple arrows represent as the lipid metabolism flow, green arrows represent as the glucose metabolism flow; orange arrows represent as the protein metabolism, black arrows represent as the ATP produced from the acetyl-CoA; the red arrows represent as the changes in metabolic pathway.

In the present study, the whole body protein content increased in the low-carnitine group. Correspondingly, the mRNA expression of GLUD1a, an enzyme involved in amino acid degradation, and mTOR, a key regulatory receptor in protein synthesis, were decreased and increased, respectively, in the mildronate group. These findings indicate that, carnitine depletion-related inhibition of lipid degradation may promote protein synthesis, but the underlying mechanism is unclear. A full account of the interactions between lipid, glucose, and protein metabolism in the low-carnitine model zebrafish is provided in **Figure 7**.

## Conclusion

This is the first trial to illustrate the altered nutrient metabolism in the fish with inhibited carnitine synthesis. The decreased hepatic concentration of carnitine, which was caused by mildronate treatment, inhibited mitochondrial FA β-oxidation, resulted in lipid accumulation in liver, and also led to a compensatory increase in glucose utilization and changes in protein metabolism. These results confirm the key roles of mitochondrial fatty acid β-oxidation in nutrient metabolism in fish, and also provide new aspects to understand the mechanisms of some metabolic diseases, such as fatty liver, in farmed fish. Moreover, the present study also established a low-carnitine fish model for future physiological studies.

## Author Contributions

Z-YD, D-LL, and J-ML designed the research. J-ML, L-YL, and XQ conducted the research. J-ML, D-LL, XW, and M-LZ analyzed the data. PD, LD, and SL contributed to the final writing of the paper. J-ML and Z-YD wrote the manuscript. Z-YD had primary responsibility for the final content. All authors read and approved the final manuscript.

## Conflict of Interest Statement

The authors declare that the research was conducted in the absence of any commercial or financial relationships that could be construed as a potential conflict of interest.

## Supplementary Material

FIGURE S1 The effects of different doses of mildronate on the expressions of the genes related detoxification and inflammation after 3 weeks. **(A)** Detoxification related genes; **(B)** inflammation related genes. All values are means ± SEM (*n* = 6). The values that are not followed by the same letter are significantly different (*P* < 0.05). *cyp1*, cytochrome P450 enzyme 1; *hsp70*, heat shock protein 70; *mif*, macrophage migration inhibitory factor; *csf*, colony stimulating factor.

FIGURE S2 The effects of dietary mildronate with 500 mg/kg BW/d on the expressions of the genes related detoxification, apoptosis and inflammation after 7 weeks. **(A)** Detoxification and apoptosis related genes; **(B)** inflammation related genes. All values are means ± SEM (*n* = 6). No significant differences were found in these markers. *cyp1*, cytochrome P450 enzyme 1; *hsp70*, heat shock protein 70; *casp3*, caspase 3; *mif*, macrophage migration inhibitory factor; *tgfb1*, transforming growth factor β1; *il1b*, interleukin 1 beta; *tnfα*, tumor necrosis factor α; *csf*, colony stimulating factor; ifn-γ, interferon γ.

TABLE S1 Formulation of the basic diet and wheat flour-dough particles.

TABLE S2 The primers used in the experiment.
