# Supplementary figures and images for: Inhibited Carnitine Synthesis Causes Systemic Alteration of Nutrient Metabolism in Zebrafish
Source: Front Physiol. 2018 May 9;9:509. doi: 10.3389/fphys.2018.00509 (PMC5954090; doi:10.3389/fphys.2018.00509)

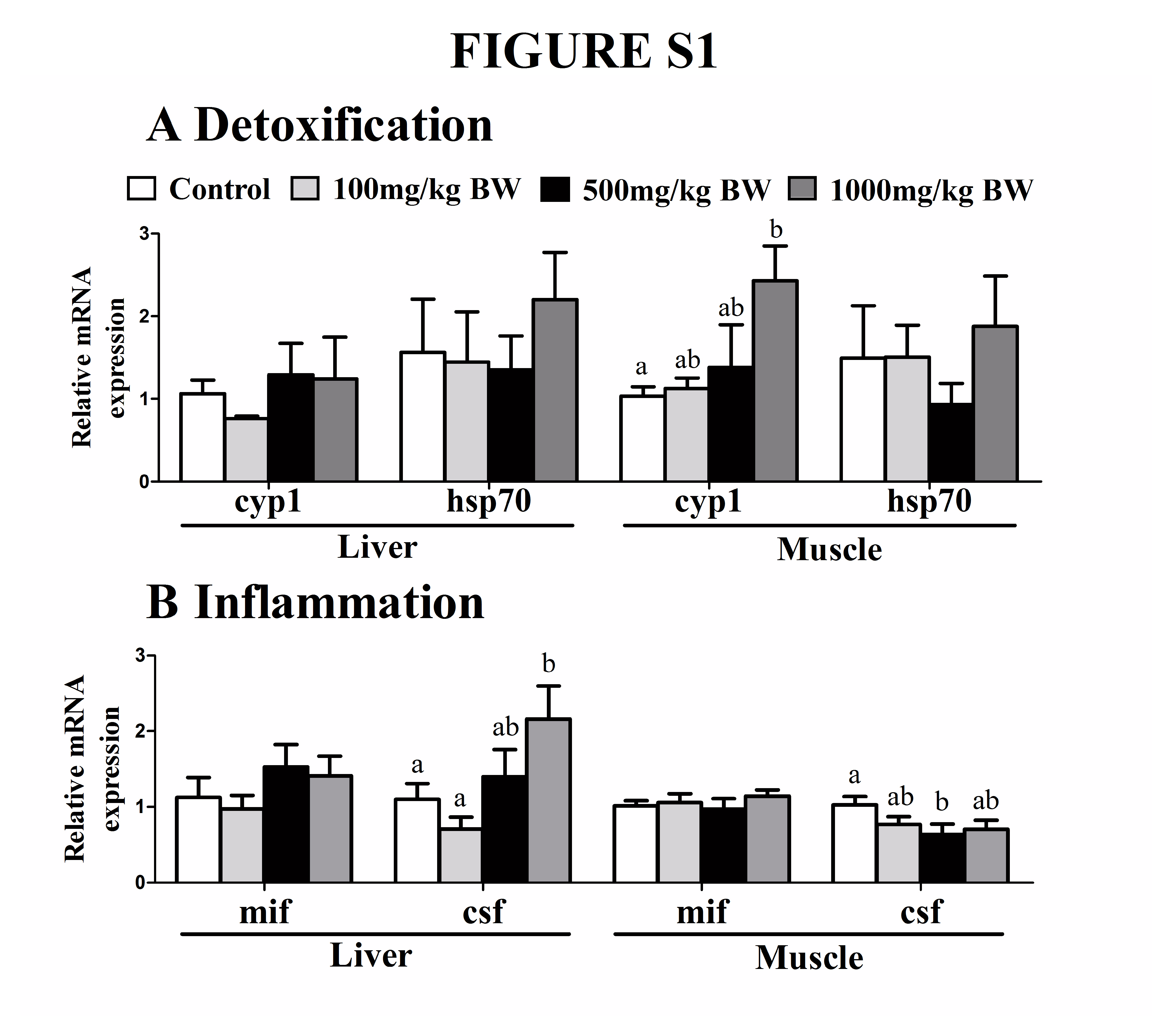

Supplement: FIGURE S1 — The effects of different doses of mildronate on the expressions of the genes related detoxification and inflammation after 3 weeks. (A) Detoxification related genes; (B) inflammation related genes. All values are means ± SEM (n = 6). The values that are not followed by the same letter are significantly different (P < 0.05). cyp1, cytochrome P450 enzyme 1; hsp70, heat shock protein 70; mif, macrophage migration inhibitory factor; csf, colony stimulating factor. [file Image_1.JPEG]

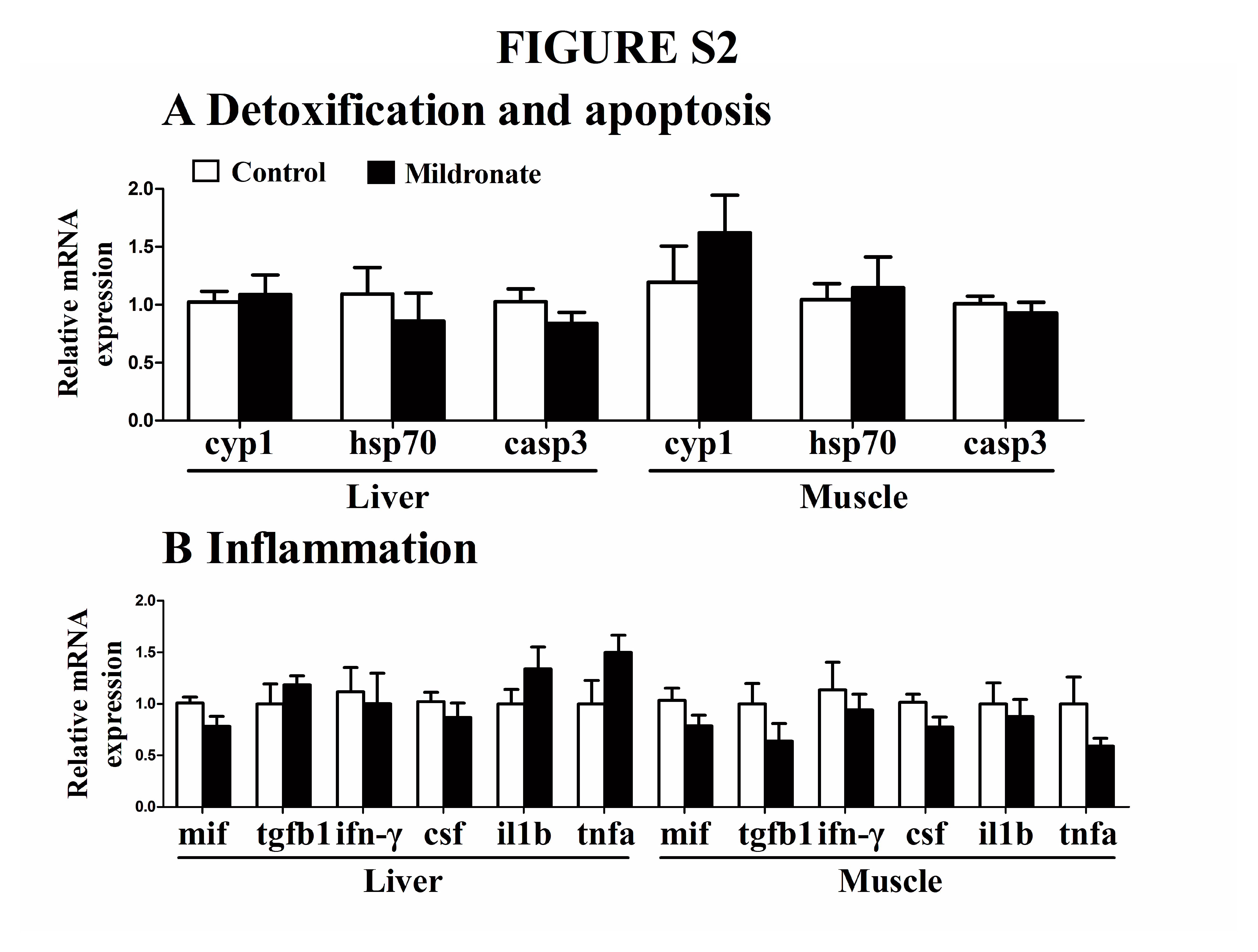

Supplement: FIGURE S2 — The effects of dietary mildronate with 500 mg/kg BW/d on the expressions of the genes related detoxification, apoptosis and inflammation after 7 weeks. (A) Detoxification and apoptosis related genes; (B) inflammation related genes. All values are means ± SEM (n = 6). No significant differences were found in these markers. cyp1, cytochrome P450 enzyme 1; hsp70, heat shock protein 70; casp3, caspase 3; mif, macrophage migration inhibitory factor; tgfb1, transforming growth factor β1; il1b, interleukin 1 beta; tnfα, tumor necrosis factor α; csf, colony stimulating factor; ifn-γ, interferon γ. [file Image_2.JPEG]
