# Supplementary material for: Inhibited Carnitine Synthesis Causes Systemic Alteration of Nutrient Metabolism in Zebrafish
Source: Front Physiol. 2018 May 9;9:509. doi: 10.3389/fphys.2018.00509 (PMC5954090; doi:10.3389/fphys.2018.00509)
Supplement: TABLE S1 — Formulation of the basic diet and wheat flour-dough particles. [file Table_1.DOC]

**Table S1.** Formulation of the basic diet and wheat flour-dough particles

| *Diet component(g/kg)* |  |
| --- | --- |
| Casein | 280 |
| Gelatin | 70 |
| Soybean oil | 70 |
| Corn starch | 414.75 |
| Vitamin premix^1^ | 15 |
| Mineral premix^2^ | 45 |
| CMC | 30 |
| Cellulose | 70 |
| Choline chloride | 5 |
| BHT | 0.25 |
| Total protein | 350 |
| Total lipid | 70 |
| Total glycogen | 414.75 |
| *Nutrient composition* |  |
| Dry matter(%) | 92.32 |
| Crude protein(%) | 34.59 |
| Crude lipid(%) | 6.89 |
| *Wheat flour component(g/kg)* |  |
| Protein(g) | 122 |
| Lipid(g) | 15 |
| Carbohydrate(g) | 700 |

^1^ Vitamin premix, (mg or IU/kg): 500,000 I.U. (international units) Vitamin A, 50,000 I.U. Vitamin D3, 2500 mg Vitamin E, 1000 mg Vitamin K3, 5000 mg Vitamin B1, 5000 mg Vitamin B2, 5000 mg Vitamin B6, 5000 μg Vitamin B12, 25,000 mg Inositol, 10,000 mg Pantothenic acid, 100,000 mg Cholin, 25,000 mg Niacin, 1000 mg Folic acid, 250 mg Biotin, 10,000 mg Vitamin C.

^2^ Mineral premix, (g/kg): 314.0 g CaCO_3_; 469.3 KH_2_PO_4_; 147.4 g MgSO_4_·7H_2_O; 49.8 g NaCl; 10.9 g Fe(II) gluconate; 3.12 g MnSO_4_·H_2_O; 4.67 g ZnSO_4_·7H_2_O; 0.62 g CuSO_4_·5H_2_O; 0.16 g KJ; 0.08 g CoCl_2_·6H_2_O; 0.06 g NH_4_ molybdate; 0.02 g NaSeO_3_.

**Table S2. The primers used in the experiment**

| **Gene name** | **Primers (5’-3’)** | **GenBank NO.** |
| --- | --- | --- |
| *ef1α* | F: CCCCTGGACACAGAGACTTCATC | L23807.1 |
|  | R: ATACCAGCCTCAAACTCACCGAC |  |
| *β-actin* | F: TCTGGTGATGGTGTGACCCA  R: GGTGAAGCTGTAGCCACGCT | AY222742 |
| *bbox1* | F: CCCATGGCTAACAATGTTGCCTA | NM_001017717.1 |
|  | R: ATCAGCCTGACGGACACAATGTA |  |
| *cpt1 (liver)* | F: CATCCTTAGGCCTGCTCTTCAAA | NM_001044854 |
|  | R: ACCATGACACCCCCAACTAACAT |  |
| *cpt1 (muscle)* | F: CCTCCATGGGCACGATTGATAA | NM_001005940.1 |
|  | R: GCAAACAGGATGGCACTCAACA |  |
| *ehhadh* | F: GAATACTTGTGAGGTGGCTCTGGA | NM_207068.1 |
|  | R: AGGACACGGTGTGGTCAGCAT |  |
| *acox* | F: TGGAAGGACATGATGCGCTTT | NM_213147.1 |
|  | R: AGGCTGCCGGGCAAAAA |  |
| *acc* | F: GCGTGGCCGAACAATGGCAG | NM_001271308.1 |
|  | R: GCAGGTCCAGCTTCCCTGCG |  |
| *fasn* | F: GGAGCAGGCTGCCTCTGTGC | XM_009306806.1 |
|  | R: TTGCGGCCTGTCCCACTCCT |  |
| *dgat2* | F: ACGCATAACCTGCTTCCC | NM_001030196.1 |
|  | R: TCCTGTGGCTTCTGTCCC |  |
| *lpl* | F: ACATTTCCTCGGGATTGGAAACT | NM_131127.1 |
|  | R: TCCATCATCCATTCTGTGGCAT |  |
| *cd36* | F:TGAACAAAATCAAGGAGCACACAA | NM_001002363.1 |
|  | R:ATCCGGGAAATCAGCTCATTCTT |  |
| *fabp4* | F:TCAACGAGCCGTTCGAGGA | AY628221.2 |
|  | R:TCCCAGGTCTGTTTCTGCACAA |  |
| *atgl* | F: GCGTGACGGATGGAGAAA | KP325485.1 |
|  | R: AGGCCACAGTAAACAGGAATAT |  |
| *hsl* | F: CGGCAAGGACAGGACAGT | NM_001316725.1 |
|  | R: GCATGGAGAAAGAGGAGCT |  |
| *mao* | F: AACGCATACGACGTGATCGTGAT | NM_212827 |
|  | G: TTCCAGGACCACAGGATTCAGG |  |
| *cat* | F: TCGACGGATCAGATGAAACTGTG | NM_130912 |
|  | G: CGCATTTAACTTGTCCCCTATCG |  |
| *insra* | F: TTGTGATGGAGGGAGGATATCTGG | NM_001142672.1 |
|  | R: GGGCCGCATTTTGGGATTAT |  |
| *insrb* | F: TTTCGCCTACATCTTGTGCCTCT | NM_001123229.1 |
|  | R: AGTTCTCCAAAACCCGCAGGTT |  |
| *pfk (liver)* | F: GTAACACGCATGGGCATTTTTG | NM_001017596 |
|  | R: TCGCCAGTTTGATGTGATCTCCT |  |
| *pfk (muscle)* | F: ATCACATCCGTCCTGCTACATGG | NM_001004575 |
|  | R: TGGTCTGGAAATCCTTACAGCG |  |
| *pk (liver)* | F: ATCACTGCCCGCAACACCA | NM_201289.1 |
|  | R: TCATTCCTGCTTTCACCATCTCC |  |
| *pk (muscle)* | F: TGAACATCGCTCGCATGAACTT | NM_199333.1 |
|  | R: TCAAAGCTGGCACAAGCTTCA |  |
| *pck1* | F: ATCGCATCACGCATCGCTAAA | NM_214751 |
|  | R: CCGCTGCGAAATACTTCTTCTGT |  |
| *g6p* | F: TGGCAGTGATAGGAGATTGGCTT | BC148168.1 |
|  | R: AGTAGGACGTCTCATGGACCCAC |  |
| *gys (muscle)* | F: GGCACTCAGGAGAACCATTGATAA | NM_201180.1 |
|  | R: TCCAGCAGAACCACATATGGTGA |  |
| *gys (liver)* | F: TTGAAGATCTCCTGCTCTTTGAGG | NM_001018679.1 |
|  | R: CATTCGTCCACAGTGATCTTTGCT |  |
| *apn* | F: GGTGGCTTTTACCGGAGTGAATA | XM_001920383.5 |
|  | R: CAAGGAAATGCTTTTCTGGCATC |  |
| *pept1* | F: TGGTGAATGAGTTCTGTGAGCGA | AY300011.1 |
|  | R: ACAGGTCATCATCCCAACCAATG |  |
| *glud1a* | F: AGGACATTGTGCATTCGGGATT | NM_212576 |
|  | R: CCTCAGATCCAGCCCAAGGTTAT |  |
| *glud1b* | F: GATGTCCTGGATTGCTGACACCT | NM_199545 |
|  | R: CCACCCTGGCTAATGGGTTTT |  |
| *mtor* | F: TGGGAGCAGACAGGAATGAAGG | NM_001077211.2 |
|  | R: TGCACCTGCTGGAAAAAGAATG |  |
| *cyp1* | F:ATTCATGAAGAGGCTGGTGATGG | NM_131879.2 |
|  | R:TTTTCGGTCTTCGCAGTGGTT |  |
| *hsp70* | F:ACCAGGGCAACAGAACAACA | NM_001113589 |
|  | R:GTCATCAAACCTCCTGCCGA |  |
| *casp3* | F:GAGACCGCTGCCCATCACT | NM_131877.3 |
|  | R:GCATCCTTTCACGACCATCTG |  |
| *mif* | F:TGGGTTTACTCAACAAACACCTCG | NM_001043321.1 |
|  | R:TCCAAAGGTGCTGTTGTTCCAG |  |
| *ifn-γ* | F:CAGAGCTCAGGACGTATGCAGAAA | NM_212864.1 |
|  | R:AGACACGCTTCAGCTCAAACAAAG |  |
| *csf* | F:AACTGCAGGCTGACATTCAAGAAC | NM_001145242.1 |
|  | R:TCTGGGACTGCTCTTCTGATGTCT |  |
| *il1b* | F:ATGATGGCATGCGGGCAATAT | NM_212844.2 |
|  | R:AGCGGATCTGAACAGTCCATCTC |  |
| *tgfb1* | F:ACTACTTTGGCAAGGAGGTGCAT | NM_182873.1 |
|  | R:CATCTCGGACACGTTGAAAAACA |  |
| *tnfα* | F:TCTGCTTCACGCTCCATAAGACC | NM_212859.2 |
|  | R:GCCTTGGAAGTGAAATTGCCTT |  |
